# Elevated AST/ALT ratio is associated with all-cause mortality in patients with stable coronary artery disease: a secondary analysis based on a retrospective cohort study

**DOI:** 10.1038/s41598-022-13355-2

**Published:** 2022-06-02

**Authors:** Xiaobo Liu, Peng Liu

**Affiliations:** 1 The Affiliated Hospital of Weifang Medical College, Shandong, China; 2 Department of Anatomy, Guangxi Medical University, Nanning, 530021 Guangxi China

**Keywords:** Cardiology, Cardiac device therapy, Cardiovascular biology, Interventional cardiology

## Abstract

The aim of this study is to explore the association between the aspartate amino transferase (AST)/alanine aminotransferase (ALT) ratio and all-cause mortality (ACM) in stable coronary artery disease (CAD) patients treated by percutaneous coronary intervention (PCI). The study is a secondary analysis of a retrospective cohort study involving 203 stable CAD patients. Patients were divided into two groups, based on the optimal AST/ALT ratio threshold calculated by the ROC curve (low group: AST/ALT ratio < 1.40; high group: AST/ALT ratio ≥ 1.40). Results were compared using hazard ratio (HR) and a 95% confidence interval (CI). ACM occurred in 18 patients after an average follow-up time of 749 (435–1122) days. Among them, ACM occurred in 6 patients in the low group and 12 patients in the high group, with significant differences between the groups (4.65% versus 16.22%, P value = 0.005). In the Kaplan–Meier analysis, an elevated AST/ALT ratio was associated with increased ACM in stable ACD patients (HR 3.78, 95% CI 1.44–9.93, P value < 0.001). An elevated AST/ALT ratio was still found to be an independent prognostic factor for ACM (HR 2.93, 95% CI 1.08–7.91, P value = 0.034) after adjusting for potential confounders. Therefore, an elevated AST/ALT ratio is an independent prognostic factor for ACM in stable ACD patients.

## Introduction

Stable coronary artery disease (CAD) is the most common clinical manifestation of ischemic heart disease and one of the leading causes of death worldwide^1–3^. Coronary revascularization is the main treatment for both symptomatic patients and ischemic coronary lesions, and significantly reduces ischemia and adverse event incidence, compared with drug therapy use alone^1,4,5^. Percutaneous coronary intervention (PCI) has been proven to be a feasible, safe, and effective treatment for symptomatic patients with stable CAD^6–9^. Previous studies demonstrate that the majority of stable CAD patients underwent PCI treatment and experienced lower rates of mortality, myocardial infarction (MI), and revascularization^6,7^. However, it is important to emphasize that although coronary revascularization significantly improves the outcome and quality of life of stable CAD patients, some individuals are still at a higher risk of coronary restenosis and death. A study involving 5286 stable ACD patients demonstrated that after an average follow-up time of 5 years, 6.5% of patients receiving combined PCI and medication treatment suffered a fatal event, 9.2% experienced a nonfatal myocardial infarction, 18.3% required unplanned revascularization, and around 20% patients suffered angina^10^. The above information suggests that significant heterogeneity among patients may exist, which necessitates further refinement of the risk stratification for stable ACD patients, to further improve their short and long-term prognoses.

The aspartate aminotransferase (AST)/alanine aminotransferase (ALT) ratio, previously considered as a serological parameter for identifying the degree of severity of hepatic fibrosis^11–13^, is also associated with the prognosis of a variety of diseases^14,15^, including cardiovascular disease^16,17^. A study involving 105 participants revealed that the AST/ALT ratio is inversely associated with left ventricular ejection fraction (LVEF) in patients with chronic heart failure, and may help in predicting the severity of cardiac function^16^. Another study discovered a strong association between an AST/ALT ratio ≥ 2.0 and total coronary artery occlusion, irrelevant of age, suggesting that this novel parameter could be used to predict coronary artery occlusion severity^17^. However, any association between the AST/ALT ratio and prognosis of stable CAD patients is still unclear, as relevant research is lacking. Consequently, this study aims to investigate the association between the AST/ALT ratio and all-cause mortality (ACM) in stable CAD patients.

## Materials and method

### Study population

This study is a secondary analysis of a retrospective cohort study^18^. All data used in this study was downloaded from the Dryad Digital Repository (https://datadryad.org/). The Dryad Digital Repository is a non-profit website, supported by the National Science Foundation of the United States^19^. It is a data platform that stores the raw data behind scientific publications, for reuse and reference. Using the Dryad Digital Repository dataset, researchers can access raw data from different studies, check and validate published results, or test newly proposed hypotheses by performing novel statistical analysis^20^. The data used in this study is a secondary analysis of Suzuki’s study^18^ (Title: Prognostic significance of serum albumin in patients with stable coronary artery disease treated by percutaneous coronary intervention. https://datadryad.org/stash/dataset/doi:10.5061/dryad.fn6730j). In short, Suzuki’s study^18^ is a retrospective cohort study conducted at Shinonoi General Hospital. Patients newly diagnosed with stable CAD between October 2014 and October 2017 (patients with previously diagnosed myocardial infarction or malignant tumors were excluded) were recruited, and all patients underwent elective PCI treatment. Suzuki’s study^18^ received approval from the Shinonoi General Hospital Ethics Committee and obtained written informed patient consent. The study was performed according to the Helsinki Declaration.

### Data collection

Data was collected upon admission. Data gathered included clinical characteristics, previous history, CAD risk factors, comorbidities, laboratory results, echocardiography, angiographic features, medication used after discharge, and post-discharge follow-up outcomes, as previously described in Suzuki’s study^18^. All provided data is completely anonymous.

### Definitions and endpoints

As described in Suzuki’s study^18^, therapeutic clinicians use all available information, including symptoms, laboratory results, electrocardiograms, echocardiography, and coronary angiography, to accurately diagnose old myocardial infarction. Stable CAD was defined as having ≥ 90% angiographic evidence of epicardial coronary artery stenosis, or ≥ 75% angiographic evidence of epicardial coronary artery stenosis. Occurrence of chest pain symptoms or stress ischemia induced by exercise, or any clinical stress test pattern is also included. Patients were given aspirin and thienopyridines pre-operation. Cardiologists performed coronary angiography and PCI according to standard protocols and guidelines. All-cause mortality (ACM) is the primary endpoint in this study.

### Statistical analysis

Firstly, since the goal of this study is to explore the association between the AST/ALT ratio and ACM in stable CAD patients, the normality of the AST/ALT ratio is initially assessed via a histogram. Secondly, the receiver operating characteristic (ROC) curve was used to explore the diagnostic value and optimal threshold of the AST/ALT ratio for ACM. The patients were subsequently divided into two groups according to the optimal threshold. Meanwhile, the sensitivity, specificity, and Youden index of the AST/ALT ratio in diagnosing ACM were recorded.

Continuous variables were represented by the mean and standard deviation (SD), and differences between groups were compared by an independent sample t-test. Categorical variables were represented by cases and percentages, and differences between groups were compared via chi-squared test. Kaplan–Meier analysis, univariate, and multivariate Cox proportional hazard regression analysis were also used to estimate the influence of the AST/ALT ratio on ACM. Results were compared using hazard ratio (HR) and a 95% confidence interval (CI).

In this study, the AST/ALT ratio was considered as the independent variable, ACM was considered as the dependent variable, and other variables were considered as covariates. The following approaches were used to screen covariates. Firstly, univariate Cox proportional hazard regression analysis was used. Factors with a P-value of < 0.05 indicate that this covariate may influence the results. Secondly, if the effect of covariates on the AST/ALT ratio regression coefficient is less than 10%, those covariates are eliminated from multivariate Cox proportional hazard regression analysis^21^. Otherwise, the covariates are included in the subsequent analysis. Thirdly, considering the available case of events, the covariates to be included are carefully selected to ensure the parsimony of the ultimate model^22,23^. Furthermore, considering the possibility of multicollinearity among variables^24^, Pearson correlation analysis was performed to identify the correlation between variables. If a variable is highly correlated with the AST/ALT ratio, those covariates are excluded from the Cox proportional hazard regression analysis^25^.

In this study, IBM SPSS Statistics V22.0 (https://www.ibm.com/products/spss-statistics), R version 4.2.0 (https://www.r-project.org/), and MedCalc 20.1.4 software (https://www.medcalc.org/) are used for statistical analysis, and P < 0.05 (two-sided) indicates that there is a statistical difference between groups.

## Results

### AST/ALT ratio distribution

**Figure 1:**
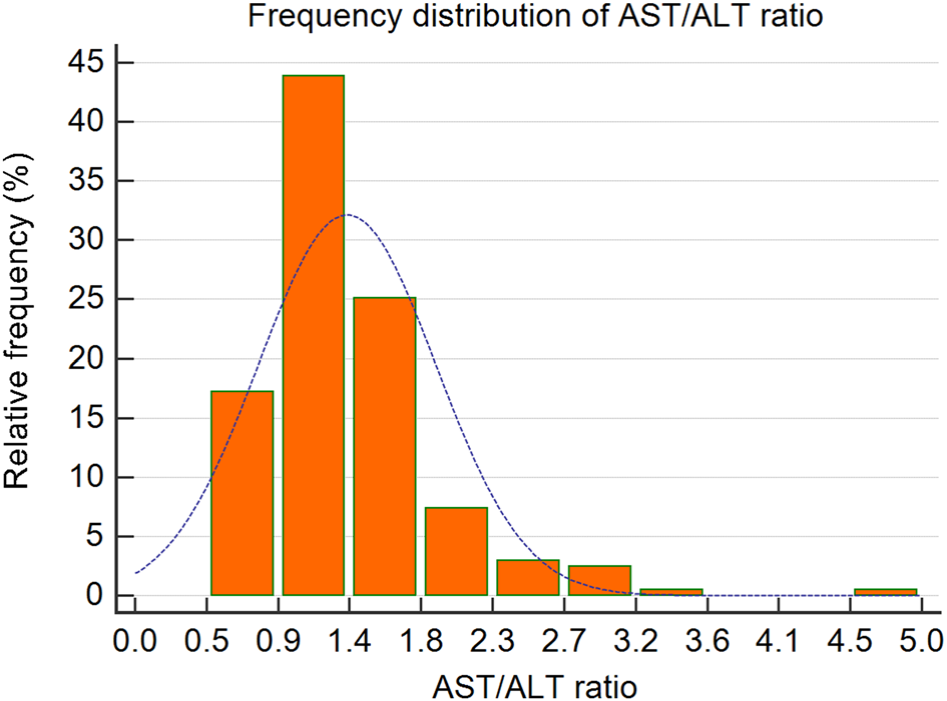
Distribution of aspartate amino transferase (AST)/alanine aminotransferase (ALT) ratio.

AST/ALT was missed in one patient, and the corresponding AST/ALT ratio could not be calculated therefore the patient was excluded from this study. 203 patients were actually included. The histogram in Fig. 1 indicates that AST/ALT ratio conforms to a skewness distribution. The AST/ALT ratio median, minimum, and maximum are 1.23, 0.52, and 4.80, respectively.

### Optimal AST/ALT ratio threshold for predicting ACM

**Figure 2:**
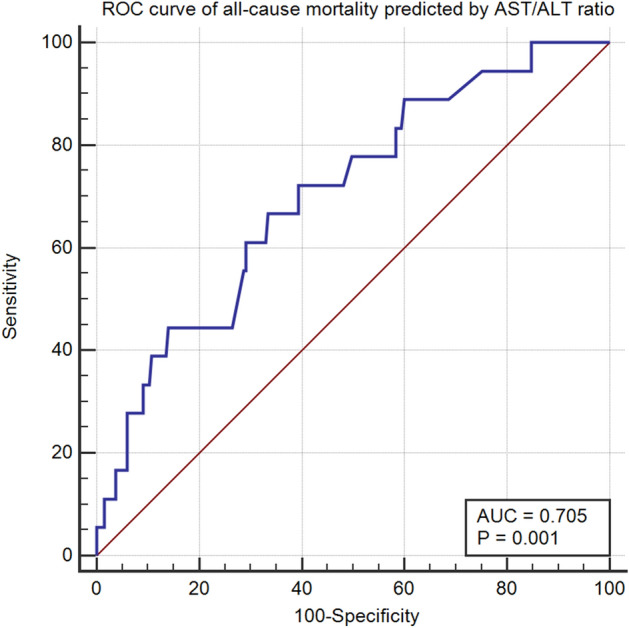
Optimal threshold of aspartate amino transferase (AST)/alanine aminotransferase (ALT) ratio for predicting all-cause mortality.

The ROC curve shows that the AST/ALT ratio could be used to predict ACM. The optimal threshold and area under the curve (AUC) are 1.40, 0.705 (95% CI 0.637 to 0.766, P value = 0.001), respectively (Fig. 2). Additionally, the Youden index, sensitivity, and specificity of the AST/ALT ratio are 0.33, 66.67%, and 66.49%, respectively.

### Study population

**Figure 3:**
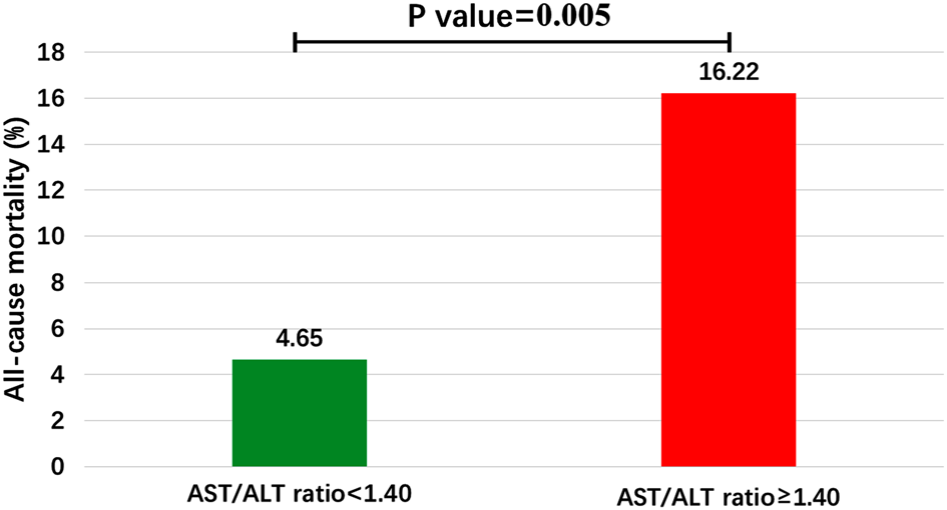
Comparison of all-cause mortality between low group (AST/ALT ratio < 1.40) and high group (AST/ALT ratio ≥ 1.40).

Patients were divided into two groups, based on the optimal threshold (1.40) of the AST/ALT ratio: low group (AST/ALT ratio < 1.40, N = 129) and high group (AST/ALT ratio ≥ 1.40, N = 74). The baseline characteristics of both groups are shown in Table 1. As also shown in Table 1, patients in the high group were older and had lower hemoglobin, albumin, estimated glomerular filtration rate (eGFR), triglycerides, and statin use, compared with the low group. On the other hand, the proportion of patients with a normal body mass index (BMI) was higher in the high group than in the low group. No significant differences in other variables were observed between the two groups. ACM occurred in 18 patients after an average follow-up time of 749 (435–1122) days. Among them, ACM occurred in 6 patients in the low group and 12 patients in the high group, with significant differences between groups (4.65% versus 16.22%, P value = 0.005, Fig. 3).

**Table 1:** Baseline characteristics of the two groups.

Variables	Low group	High group	P-value
N	129	74	
Range of AST/ALT ratio	AST/ALT ratio < 1.40	AST/ALT ratio ≥ 1.40	
Age ≥ 70 years	67 (51.93%)	57 (77.03%)	< 0.001
Male (n, %)	91 (70.54%)	50 (67.57%)	0.658
Normal BMI (18.5 ≤ BMI < 25 kg/m^2^)	66 (51.16%)	53 (71.62%)	0.004
Past smoking (n, %)	70 (54.26%)	30 (40.54%)	0.060
Systolic blood pressure (mmHg)	137.22 ± 20.43	134.74 ± 20.62	0.407
Diastolic blood pressure (mmHg)	78.50 ± 12.77	75.55 ± 13.91	0.128
LVEF (%)	63.85 ± 9.29	62.11 ± 10.56	0.225
**Laboratory data**			
Hemoglobin ≥ 12 g/dL	113 (87.59%)	52 (70.27%)	0.002
Albumin ≥ 3.2 g/dL	122 (94.57%)	61 (82.43%)	0.005
eGFR (mL/min/1.73 m^2^)	64.80 ± 23.37	54.95 ± 25.64	0.006
AST/ALT ratio	1.04 ± 0.22	1.89 ± 0.57	< 0.001
Total cholesterol (mg/dL)	188.28 ± 36.13	180.40 ± 34.65	0.166
Triglycerides (mg/dL)	145.13 ± 114.36	116.03 ± 56.48	0.045
HDL (mg/dL)	49.70 ± 12.24	50.96 ± 14.79	0.521
LDL (mg/dL)	112.46 ± 28.48	106.07 ± 28.50	0.132
HbA1c (%)	7.96 ± 16.97	6.17 ± 0.98	0.386
CRP (mg/dL)	0.41 ± 0.94	0.52 ± 1.24	0.508
**Previous diseases**			
Old cerebral infarction (n, %)	21 (16.28%)	13 (17.57%)	0.813
Peripheral artery disease (n, %)	35 (27.13%)	18 (24.32%)	0.661
Atrial fibrillation (n, %)	15 (11.63%)	11 (14.86%)	0.507
Hypertension (n, %)	99 (76.74%)	51 (68.92%)	0.222
Diabetes mellitus (n, %)	48 (37.21%)	24 (32.43%)	0.494
**Medication**			
Aspirin (n, %)	128 (99.22%)	73 (98.65%)	0.689
Thienopyridines (n, %)	126 (97.67%)	73 (98.65%)	0.631
Warfarin (n, %)	2 (1.55%)	3 (4.05%)	0.268
DOAC (n, %)	13 (10.08%)	8 (10.81%)	0.869
Ezetimibe (n, %)	2 (1.55%)	1 (1.35%)	0.910
PPI (n, %)	88 (68.22%)	45 (60.81%)	0.285
Statins (n, %)	80 (62.02%)	30 (40.54%)	0.003
ACEI (n, %)	12 (9.30%)	7 (9.46%)	0.970
ARB (n, %)	54 (41.86%)	34 (45.95%)	0.572
Beta-blocker (n, %)	35 (27.13%)	20 (27.03%)	0.987
MRA (n, %)	7 (5.43%)	4 (5.41%)	0.995
**Lesional characteristics**			
Multivessel disease (n, %)	35 (27.13%)	17 (22.97%)	0.514
Bifurcation lesions (n, %)	65 (50.39%)	36 (48.65%)	0.811
LMT lesions (n, %)	10 (7.75%)	3 (4.05%)	0.300
Ostial lesions (n, %)	19 (14.73%)	10 (13.51%)	0.812
Calcified lesions (%)	16 (12.40%)	13 (17.57%)	0.312
CTO lesions (n, %)	10 (7.75%)	2 (2.70%)	0.142
All-cause death (n, %)	6 (4.65%)	12 (16.22%)	0.005

Continuous variables are described by mean and standard deviation, and categorical variables are described by number of cases and percentage.

*BMI* body mass index, *eGFR* estimated glomerular filtration rate, *HDL* high density lipoprotein, *LDL* low density lipoprotein, *HbA1c* hemoglobin A1c, *CRP* C-reactive protein, *LVEF* left ventricular ejection fraction, *DOAC* direct oral anticoagulants, *PPI* proton pump inhibitor, *ACEI* angiotensin-converting enzyme inhibitor, *ARB* angiotensin-receptor blocker, *MRA* mineralocorticoid receptor antagonist, *MACE* major adverse cardiac events, *CTO* chronic total occlusion, *DES* drug-eluting stent, *LMT* left main trunk.

### Univariate Cox proportional hazard regression analysis associated with ACM

In Table 2, univariate Cox proportional hazard regression analysis indicated that advanced age (HR 5.04, 95% CI 1.16–21.96) and AST/ALT ratio (HR 3.56, 95% CI 1.33–9.51) were associated with a higher risk of ACM, while past smoking (HR 0.29, 95% CI 0.10–0.89), hemoglobin (HR 0.13, 95% CI 0.05–0.35), albumin (HR 0.16, 95% CI 0.06–0.41), eGFR (HR 0.98, 95% CI 0.96–0.99), total cholesterol (HR 0.98, 95% CI 0.96–0.99), low density lipoprotein (LDL, HR 0.98, 95% CI 0.96–0.99), aspirin (HR 0.10, 95% CI (0.01–0.79), and statins (HR 0.34, 95% CI 0.12–0.94) were associated with a lower risk of ACM. No other variables were found to be associated with ACM in the univariate analysis.

**Table 2:** Univariate Cox proportional hazard regression analysis associated with ACM.

Variables	HR (95% CI)	P value
Age ≥ 70 years	5.04 (1.16–21.96)	0.031
Male (n, %)	1.00 (0.37–2.69)	0.997
Normal BMI (18.5 ≤ BMI < 25 kg/m^2^)	0.68 (0.26–1.83)	0.450
Past smoking (n, %)	0.29 (0.10–0.89)	0.030
Systolic blood pressure (mmHg)	0.99 (0.97–1.02)	0.909
Diastolic blood pressure (mmHg)	1.00 (0.97–1.04)	0.931
LVEF (%)	0.97 (0.93–1.01)	0.119
**Laboratory data**		
Hemoglobin ≥ 12 g/dL	0.13 (0.05–0.35)	< 0.001
Albumin ≥ 3.2 g/dL	0.16 (0.06–0.41)	< 0.001
eGFR (mL/min/1.73 m^2^)	0.98 (0.96–0.99)	0.018
AST/ALT ratio ≥ 1.40	3.56 (1.33–9.51)	0.012
Total cholesterol (mg/dL)	0.98 (0.96–0.99)	0.016
Triglycerides (mg/dL)	0.99 (0.98–1.01)	0.154
HDL (mg/dL)	0.97 (0.93–1.01)	0.138
LDL (mg/dL)	0.98 (0.96–0.99)	0.039
HbA1c (%)	0.59 (0.29–1.23)	0.162
CRP (mg/dL)	1.21 (0.96–1.51)	0.103
**Previous diseases**		
Old cerebral infarction (n, %)	1.41 (0.46–4.28)	0.546
Peripheral artery disease (n, %)	2.29 (0.88–5.70)	0.088
Atrial fibrillation (n, %)	2.14 (0.69–6.57)	0.183
Hypertension (n, %)	0.87 (0.31–2.43)	0.787
Diabetes mellitus (n, %)	0.47 (0.16–1.44)	0.188
**Medication**		
Aspirin (n, %)	0.10 (0.01–0.79)	0.029
Thienopyridines (n, %)	0.39 (0.05–3.04)	0.375
Warfarin (n, %)	2.89 (0.38–21.85)	0.303
DOAC (n, %)	1.09 (0.25–4.78)	0.907
Ezetimibe (n, %)	0.05 (0.01–1654)	0.692
PPI (n, %)	0.74 (0.29–1.87)	0.523
Statins (n, %)	0.34 (0.12–0.94)	0.038
ACEI (n, %)	0.97 (0.22–4.32)	0.966
ARB (n, %)	2.19 (0.85–5.68)	0.105
Beta-blocker (n, %)	1.09 (0.39–3.06)	0.874
MRA (n, %)	2.35 (0.54–10.25)	0.255
**Lesional characteristics**		
Multivessel disease (n, %)	1.09 (0.39–3.07)	0.866
Bifurcation lesions (n, %)	1.51 (0.58–3.91)	0.393
LMT lesions (n, %)	1.49 (0.34–6.52)	0.595
Ostial lesions (n, %)	0.65 (0.15–2.90)	0.576
Calcified lesions (%)	1.90 (0.62–5.81)	0.257
CTO lesions (n, %)	0.05 (0.00–523)	0.517

Data are described by odds ratio and 95% confidence interval.

*BMI* body mass index, *eGFR* estimated glomerular filtration rate, *HDL* high density lipoprotein, *LDL* low density lipoprotein, *HbA1c* hemoglobin A1c, *CRP* C-reactive protein, *LVEF* left ventricular ejection fraction, *DOAC* direct oral anticoagulants, *PPI* proton pump inhibitor, *ACEI* angiotensin-converting enzyme inhibitor, *ARB* angiotensin-receptor blocker, *MRA* mineralocorticoid receptor antagonist, *MACE* major adverse cardiac events, *CTO* chronic total occlusion, *DES* drug-eluting stent, *LMT* left main trunk.

### Lasso regression analysis associated with ACM

**Figure 4:**
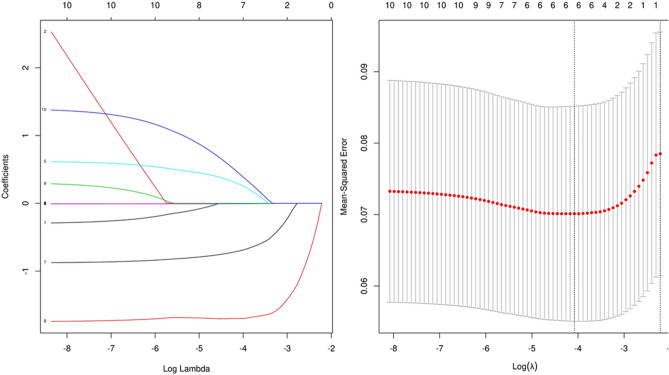
Variables associated with all-cause mortality screened using Lasso regression analysis.

Variables, including age, AST/ALT ratio, past smoking, hemoglobin, albumin, eGFR, total cholesterol, LDL, aspirin, and statins, selected from univariate Cox analysis, were incorporated into Lasso regression analysis to further screen out variables associated with ACM. The Lasso regression analysis revealed that when the minimum mean square error λ is 0.017, the model is optimal. At this time, the identified variables are age, AST/ALT ratio, hemoglobin, albumin, total cholesterol, and LDL (Fig. 4).

### Kaplan–Meier analysis

**Figure 5:**
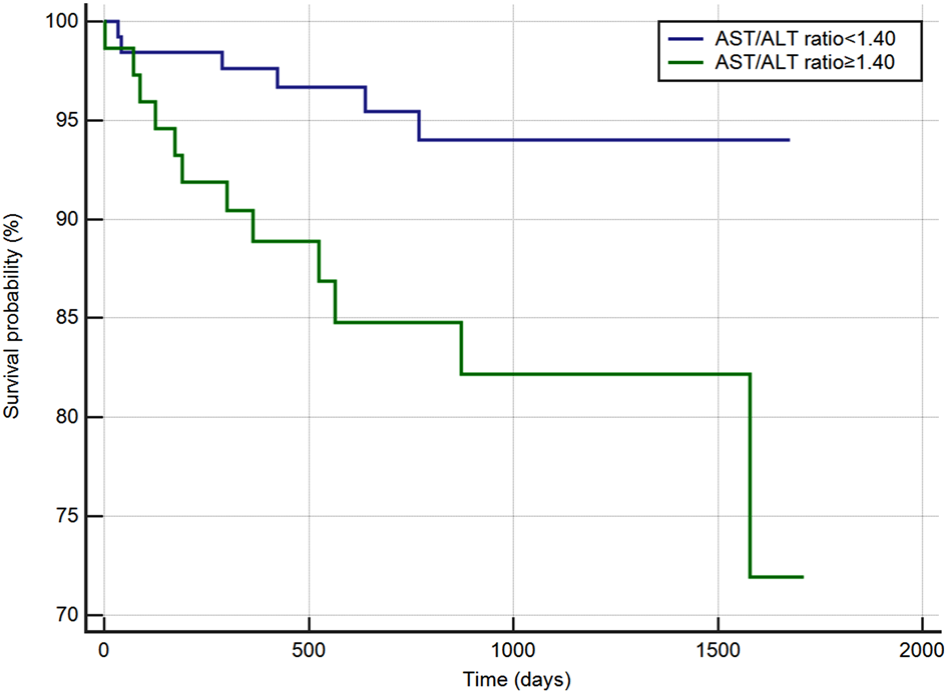
Kaplan–Meier analysis between low group (AST/ALT ratio < 1.40) and high group (AST/ALT ratio ≥ 1.40).

Kaplan–Meier analysis of the AST/ALT ratio and ACM is presented in Fig. 5. In the Kaplan–Meier analysis, an elevated AST/ALT ratio is associated with increased ACM in stable ACD patients (HR 3.78, 95% CI 1.44–9.93, P < 0.001) after an average follow-up time of 749 (435–1122) days.

### Multivariate Cox proportional hazard regression analysis

**Figure 6:**
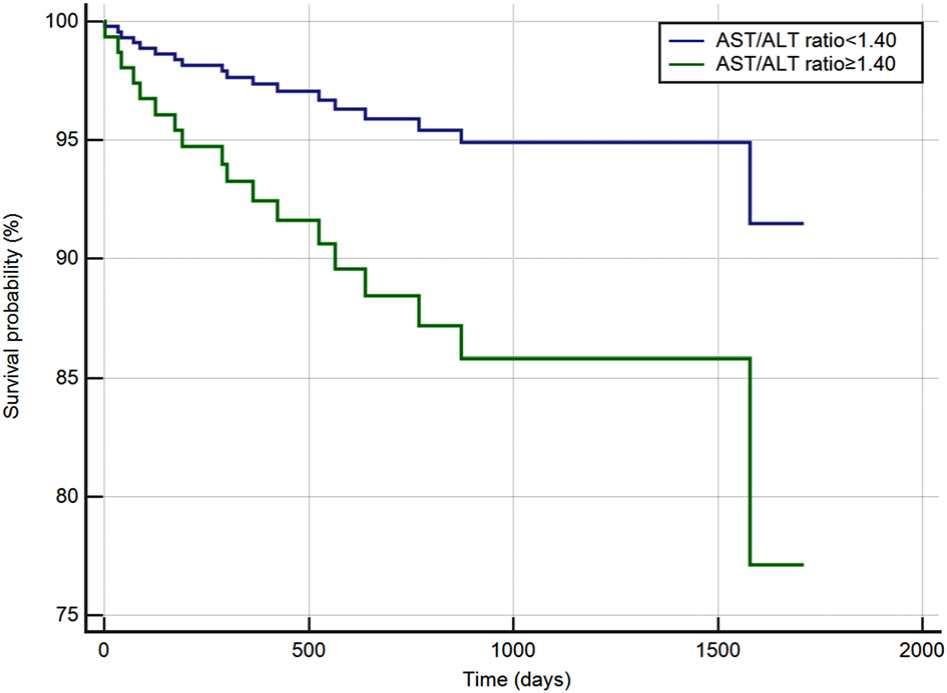
Multivariate Cox proportional hazard regression analysis of aspartate amino transferase (AST)/alanine aminotransferase (ALT) ratio and all-cause mortality.

Based on the above covariate screening criteria, the influence of age, total cholesterol, and LDL, on the AST/ALT ratio regression coefficient is less than 10%, therefore these covariables can be excluded. Furthermore, Suzuki’s^18^ study proposes that albumin is associated with the prognosis of stable CAD patients, considering the high correlation between hemoglobin and the AST/ALT ratio. Consequently, to ensure the simplicity of the model, albumin was adjusted as a confounding factor in the subsequent analysis. After adjusting for potential confounders using multivariate Cox regression analysis, an elevated AST/ALT ratio was still found to be an independent prognostic factor for ACM in stable ACD patients (HR 2.93, 95% CI 1.08–7.91, P value = 0.034, Fig. 6). This indicates that a higher AST/ALT ratio is associated with a higher risk of ACM.

## Discussion

The main findings of this retrospective cohort study, based on long-term follow-up, are as follows: firstly, to the best of our knowledge, this study is the first to use AST/ALT ratio to assess outcomes in stable CAD patients. It is found that the AST/ALT ratio is an important serological marker that can be used to predict ACM in stable CAD patients. The optimal prediction threshold is 1.4, and the sensitivity and specificity are 66.67% and 66.49%, respectively. Secondly, after adjusting for potential confounding factors, the AST/ALT ratio is a prognostic risk factor for ACM in stable CAD patients. This suggests that the AST/ALT ratio could be used in clinical practice to further refine risk stratification for stable CAD patients. Furthermore, the results of this study suggest that if the AST/ALT ratio is measured at admission, patients at high risk of ACM can be identified early. Therefore, clinicians should focus on such high-risk populations during follow-up.

It is acknowledged that the AST/ALT ratio is an important blood circulation biomarker. Most previous studies on the AST/ALT ratio propose that the AST/ALT ratio level is related to the prognosis of patients suffering from different diseases^15–17^. However, few studies have stated that the AST/ALT ratio is not related to disease^26^. A retrospective cohort study involving 380 patients with toxic hepatitis revealed that AST/ALT ratios could not predict the severity of liver fibrosis^26^. AST is found to be highly expressed in the brain, muscle, and kidney tissues, while ALT is considered to have higher liver specificity or abundant expression in liver tissues^27^. Pathological states can lead to tissue damage and higher abnormal status, resulting in a significant increase in AST level rather than ALT, making the AST/ALT ratio an attractive potential marker^28,29^. This study also found that the AST/ALT ratio was related to stable CAD patient prognosis, that is, an elevated AST/ALT ratio is associated with ACM in stable CAD patients. After adjusting for potential confounding factors using multivariate Cox regression analysis, the relationship between the AST/ALT ratio and ACM still exists (HR 2.93, 95% CI 1.08–7.91). This indicates that the AST/ALT ratio can serve as a serological marker to quantify risk stratification in stable CAD patients. Our results are consistent with previous studies regarding the AST/ALT ratio and cardiovascular disease. A large sample cohort study with 14,220 participants examined the association between the AST/ALT ratio with ACM and cardiovascular mortality in hypertensive patients. This study found that an increased AST/ALT ratio was associated with increased risk of ACM (P value < 0.05) and cardiovascular mortality (P value < 0.05) in hypertensive patients^30^, after an average follow-up time of 1.7 years. Another study, based on the general population, followed 3494 participants for up to 10 years and found that the AST/ALT ratio increased with brain natriuretic peptide (BNP). Furthermore, multivariate regression analysis suggested that a higher AST/ALT ratio was an independent predictor for ACM and cardiovascular mortality, and the authors further proposed that the AST/ALT ratio is a simple and low-cost mortality predictor^15,31^.

Possible explanations for an elevated AST/ALT ratio leading to a higher risk of ACM in stable CAD patients are as follows: previous studies have shown that high AST activity is associated with higher mortality^32,33^, and low ALT levels are associated with aging, frailty, and higher mortality, in elderly populations^34^. Furthermore, previous studies have demonstrated that the main reason for the reduction in ALT is the significant reduction in liver size and hepatic blood flow^31,35^, and patients with a history of cardiovascular disease tend to have lower ALT levels. It is speculated that low hepatic blood flow may be related to underlying myocardial injury. This phenomenon, known as cardiohepatic syndrome^36,37^, proposes that the heart and liver interact to exacerbate or slow disease progress. Considering these cardio-hepatic interactions, clinical practice should not only focus on the improvement of one particular organ but should focus on the functional improvement of all organs. Moreover, elevated transaminases in clinical practice may lead to a reduction in statin use, which may partially explain the increased risk of developing ACM.

This study has some limitations. Firstly, there may be potential bias inherent in retrospective studies. Secondly, this study is a secondary analysis based on a retrospective study, and its participants may have undetected liver, or other, disease that affects the serum AST or ALT levels, leading to possible confounding bias in the AST/ALT ratio. Thirdly, the subjects of this study were patients from a single center, and caution should be exercised when applying this result to other ethnic groups. Fourthly, the patients included in this study were Japanese, and further evidence is needed to verify whether it is applicable to other populations. Further prospective, large sample, multi-center studies are needed to validate the results of this research.

## Conclusion

An elevated AST/ALT ratio is an independent prognostic factor for ACM in stable ACD patients. In clinical practice, the risk stratification of stable CAD patients could be refined by determining the AST/ALT ratio concentration, thereby improving their short- and long-term prognoses. In addition, considering that the AST/ALT ratio is an inexpensive routine serological marker in clinical practice, it has the potential for further promotion and application.

## Data Availability

All data used in this study was download from the Dryad Digital Repository (https://datadryad.org/).
